# Seroprevalence of Human Hydatidosis Using ELISA Method in Qom Province, Central Iran

**Published:** 2012

**Authors:** A Rakhshanpour, M Fasihi Harandi, SS Moazezi, MT Rahimi, M Mohebali, GhH Mowlavi, Z Babaei, M Ariaeipour, Z Heidari, MB Rokni

**Affiliations:** 1 Zoonoses Research Center, Dept. of Medical Parasitology and Mycology, School of Medicine, Kerman University of Medical Sciences, Kerman, Iran; 2 Dept. of Medical Parasitology and Mycology, School of Public Health, Tehran University of Medical Sciences, Tehran, Iran; 3 Iranian Blood Transfusion Organization, Tehran, Iran; 4 Department of Medical Parasitology and Mycology, School of Medicine, Mazandaran University of Medical Sciences, Sari, Iran; 5 Center for Research of Endemic Parasites of Iran (CREPI), Tehran University of Medical Sciences, Tehran, Iran

**Keywords:** *Cystic Echinococcosis*, ELISA, Epidemiology, Risk factor, Iran

## Abstract

**Background:**

The objective of this study was to determine the prevalence of cystic echinococcosis (CE) in Qom Province, central Iran using ELISA test.

**Methods:**

Overall, 1564 serum samples (800 males and 764 females) were collected from selected subjects by randomized cluster sampling in 2011-2012. Sera were analyzed by ELISA test using AgB. Before sampling, a questionnaire was filled out for each case. Data were analyzed using Chi-square test and multivariate logistic regression for risk factors analysis.

**Results:**

Seropositivity was 1.6% (25 cases). Males (2.2%) showed significantly more positivity than females (0.9%) (*P*= 0.03). There was no significant association between CE seropositivity and age group, occupation, and region. Age group of 30-60 years encompassed the highest rate of positivity. The seropositivity of CE was 2.1% and 1.2% for urban and rural cases respectively. Binary logistic regression showed that males were 2.5 times at higher risk for infection than females.

**Conclusion:**

Although seroprevalence of CE is relatively low in Qom Province, yet due to the importance of the disease, all preventive measures should be taken into consideration.

## Introduction

Hydatidosis or cystic echinococcosis (CE), has largely been studied as one of the most important and serious helminthic disease throughout the world (1). The importance is not only due to the damages imposed on humans but also a vast majority of animal infections causes huge waste of animal proteins. Although the agent of the disease, i.e. *Echinococcus* *granulosus*, lodged in canids intestine but the rate of human infection due the larval form of the parasite is more or less high. The parasite is cosmopolitan and posses the second rank in consideration of helminthic diseases significance (1). The highest rate of infection is reported from Africa, China, east, and south of Europe, Mediterranean coasts, Middle East, South America and mostly in rural districts (2).

Hydatidosis has been reported in Iran via different studies on human and animals and the disease is responsible for approximately 1% of admission to surgical wards (3, 4).

To combat the disease it is indispensable to encompass comprehensive rate of the disease all through the country, so although there are documented prevalence rates in different provinces, but the puzzle should be completed exactly. To reach this point the present study was conducted in Qom Province, central Iran, through which the seroprevalence of human hydatidosis was challenged via examination of collected human sera using ELISA test.

## Materials and Methods

### Area

**Fig. 1:**
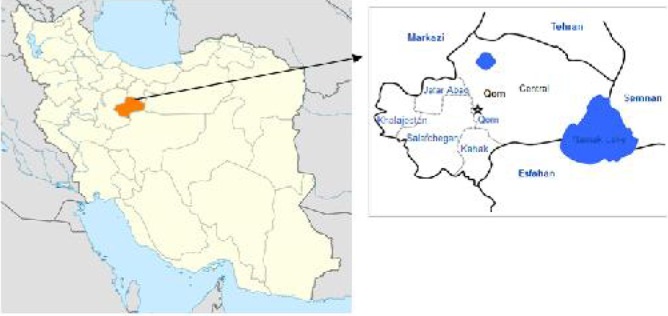
Status of Qom Province in Iran

Qom province located on the south of Tehran and central Iran is of 11238 sq. km. The province is connected to the Semnan Province in the east, to the Isfahan Province in the south and to the Markazi Province from south-west to north-west (Fig. 1). The population is estimated as one million. The province comprises of 1 city, 5 towns, 4 districts, and 936 habitations out of which 356 are populated (Source: http://amar.sci.org.ir/index_e.aspx).

### Samples

A cross-sectional study was performed on urban and rural populations of Qom Province from 2011-2012. Household data were obtained from local health authority and one random person of one family out of twenty-seven families, recommended by the statistician, was selected. Overall, 1564 serum samples (800 males and 764 females) were collected from 700 urban and 864 rural cases by randomized cluster sampling. Rural areas were as follows: Kahak, Khalajestan, Jafarieh, Salafchegan and central parts. Thirty non-infected controls from the verified bank of the laboratory were examined to set the cut-off point as 2.0 standard deviations from the mean of the seronegatives. Then the blood sample was taken from each participant and transferred to the laboratory of the Amiralmomenin Polyclinic, Qom Province. Sera were sent to Dept. of Medical Parasitology, School of Public Health, Tehran University of Medical Sciences, Iran for examination with ELISA.

A questionnaire was filled out for each individual to obtain information including the different factors correlated with the disease and the rate of people awareness of it after getting informed consent. The study was approved by Ethics Committee of the university.

### Antigen

Hydatid cyst fluid (HCF Ag) was collected from hydatid cysts in the livers and lungs of sheep slaughtered at the local abattoirs of Tehran. Antigen B was prepared and purified from HCF as described elsewhere (5).

### ELISA test

Microplate wells were coated over night at 4°C with 100 μl AgB (20 μg/ml) in 0.05 M bicarbonate buffer, pH 9.6. Wells were washed 3 times in PBS plus 0.05% Tween 20 (PBS-T) and blocked with PBS-T containing 1% BSA for 30 min at 37 °C. Sera were added at 1:500 dilutions in PBST, incubated at 37 °C for 1 h then washed as before. Antihuman IgG-HRP (Sigma Chemical Co., Poole, Dorset, United Kingdom) conjugates were added at 1: 10000 dilutions in PBS-T and the microplate incubated and washed as before. This was then developed in OPD substrate (5 mg 1, 2 phenylenediamine, 12.5 ml of 0.2 M citrate phosphate buffer pH 5, 10 μl 30% H2O2). The absorbance was read at 492 nm after 10 min using an automatic microplate reader (State Fax^®^ 2100, Awareness, USA).

### Statistical analysis

The data were analyzed using SPSS 16 and EpiInfo 6 programs. Odds ratios for risk factors analysis were calculated by multivariate logistic regression model. Only independent variables with *P* values less than 0.25 based on bivariate analysis were included in the multivariate model. *P* value less than 0.05 was considered as significant.

## Results

**Fig. 2:**
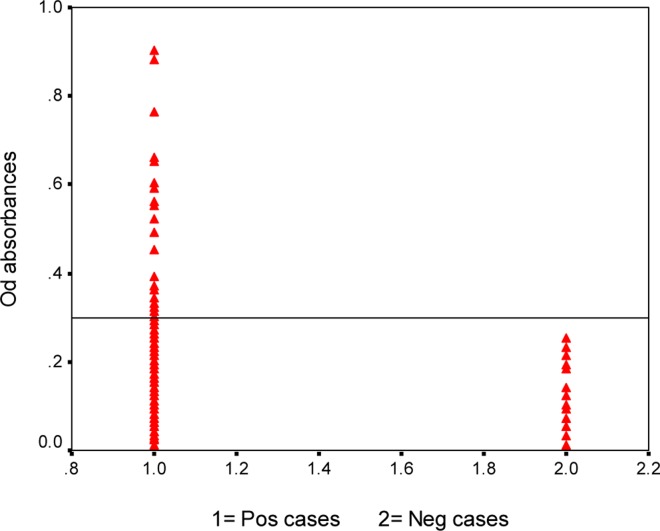
Analysis of sera from subjects and normal controls by IgG-ELISA using Antigen B. Serum samples obtained from subjects (1564, Lanes 1), and normal controls (30, Lanes 2)

Serological results showed 1.6% (twenty-five cases) seropositivity (Fig. 2). The cut-off point between clusters (set at 2.0 standard deviations from the mean of the seronegatives) was 0.3. Males (2.2%) were significantly more positive than females (0.9%) (*P*= 0.03).

There was no significant association between CE seropositivity and age group, occupation, and region. There was 1.2% seropositivity in < 30 years age group, 2.1% in 30-60 years, and 0.7% in > 60 years age group. According to occupation, employees had the highest rate of positive serology (2.4%), housewives and students 1.2% and farmers 0.5%. The prevalence of CE was 2.1% and 1.2% in urban and rural areas respectively.

Binary logistic regression showed that males were 2.5 times at higher risk to be infected than females (OR= 2.5;%95 CI= 1.03- 5.99) and multivariate logistic regression showed that males were 9.3 times at higher risk of infection than females (OR= 9.3;%95 CI = 1.44-60.12) (Table 1).

**Table 1:** Univariate and Multiple logistic regression analysis of CE seropositivity by sex, occupation, location, and knowledge

			Univariate analysis		Multivariate analysis	
Risk factor	Number	Seroprevalence % (95% CI)	Odds ratio (95% CI)	*P*-value	Odds ratio (95% CI)	*P*-value
**Sex**						
Male	800	2.2 (1.38 -3.46)	2.5(1.03-5.99)	0.04	9.3(1.44-60.12)	0.02
Female	764	0.9(0.4-1.8)	1		1	
**Occupation**						
Farmer	201	0.5(0.02-2.43)	0.2(0.03-1.55)	0.12	0.2(0.02-1.68)	0.14
Housewife	571	1.2(0.54-2.41)	0.5(0.2-1.25)	0.14	4.57(0.65-32.03)	0.13
Student	166	1.2(0.2-3.92)	0.5(0.11-2.19)	0.36	1.25(0.26-6.09)	0.78
Others	626	2.4(1.4-3.83)	1		1	
**Location**						
Urban	700	2.1(1.25-3.43)	1.87(0.83-4.19)	0.13	2.08(0.85-5.12)	0.11
Rural	864	1.2(0.59-2.05)	1		1	
**Knowledge of the disease**						
Knowledge	190	1.5(0.92-2.19)	1.83(0.68-4.93)	0.23	1.57(0.57-4.33)	0.38
Lack of knowledge	1374	2.6(0.97-5.73)	1		1	

## Discussion

In this study, we challenged the seroprevalence of human hydatidosis in Qom Province, central Iran to complete the prevalence puzzle of the disease in Iran. The study is a part of complete program to detect the situation of the disease in the country. Not mention of different aspects of elimination of each disease, knowing the exact rate of infection in a region is compulsory.

The overall prevalence of human hydatidosis in this study was determined as 1.6%. A variety of different studies within different parts of Iran shows the rate of disease from 1.2% to 21.4% based on a compendium of serological methods mainly ELISA (3). The seroprevalence of hydatidosis in humans in Golestan Province as 2.34%, Zanjan 3%, Kashan 2.4%, Meshkin Shahr 1.79%, Ilam 1.2%, Nomads of Khuzestan 13.8% and Kerman 7.3% have been reported earlier (6–12). The annual incidence rate of hydatidosis in humans has been reported in Kashan as 3/100000, Hamadan 1.33 /100,000, Babol 1.18/100,000, and throughout Iran 0.61/100,000 (8, 13–15). Accordingly, comparison the findings demonstrate more or lees a similar rate, which considering the conditions of the studied region in our study was expectable.

A valuable point of our study was using AgB of *E. granulosus*, which is considered as the most powerful antigen in terms of diagnosing the disease (16, 17). Using this antigen, we can be sure that the risk of cross-reaction has been decreased to a remarkable extent.

This survey showed that males (2.2%) were significantly more positive than females (0.9%) (*P*= 0.03). Besides, males were 2.5 times at higher risk of infection than females. This might be due to the culture of the area, where men are in more contact with risk factors than women are. An acceptable justification for this phenomenon might be the religious culture of the area, where a holy shrine is located in the city and many people come there as pilgrims, so females are preferred to work at home than outer locations. In Heidari et al. study, the highest rate of infection was in males (9).

There was no significant association between CE seropositivity and age group, occupation, and region. There was 1.2% seropositivity in < 30 years age group, 2.1% in 30-60 years, and 0.7% in > 60 years age group. In Golestan (North of Iran) and Kerman (Southeast) provinces, the highest rates of infection were in 30-60 and 20-30 years respectively (6, 12). Hydatidosis is a chronic disease of long incubation period (might be 20 to 30 yr) and accordingly a wide range of different ages is obvious in infected patients (1).

According to occupation, employees had the highest rate of positivity (2.4%), while in some other studies, housewives encompassed the highest rate of infection (3, 6, 8).

The prevalence of CE was 2.1% and 1.2% in urban and rural areas respectively. Yang et al. showed no significant association between CE seropositivity and region (18).

The present study showed that the risk of hydatidosis more or less is an issue of high importance in this region. Considering the different aspects of the disease and its remarkable side effects this finding should be regarded by local authorities. As other parasitic diseases, increasing the people awareness of the disease might be considered as the most important step in struggle with this infection.
